# Investigation of Hardness, Microstructure, and Mechanical Properties of Goat Horn Powder–Reinforced Wood-like Polyurethane Composites

**DOI:** 10.3390/polym18060723

**Published:** 2026-03-17

**Authors:** Lokman Yünlü

**Affiliations:** Department of Mechanical Engineering, Engineering Faculty, Burdur Mehmet Akif Ersoy University, 15030 Burdur, Turkey; lyunlu@mehmetakif.edu.tr

**Keywords:** wood-like polyurethane, goat horn powder, mechanical analysis, composite materials, microstructure

## Abstract

This study investigates the effect of goat horn powder (GHP) reinforcement on the hardness, microstructure, and mechanical properties of wood-like polyurethane composites. GHP, a keratin-based animal waste, was incorporated into the polyurethane matrix at weight fractions of 5, 10, 15, 20, and 25 wt.%. The mechanical behavior was evaluated through tensile, three-point bending, Charpy impact, and Shore D hardness tests, complemented by scanning electron microscopy (SEM) and energy-dispersive X-ray spectroscopy (EDS) analyses. Results indicate that GHP significantly enhances impact resistance, with 10 wt.% loading achieving a 140% improvement in impact energy compared to the neat matrix. Tensile stress improved by 12.89% at 5 wt.% loading. However, reinforcement levels exceeding 10–15 wt.% led to a decline in tensile and flexural performance due to particle agglomeration and weak interfacial adhesion. Shore D hardness increased systematically with higher GHP content across all ratios. The study demonstrates that GHP is a functional, sustainable reinforcing element that improves toughness and hardness while supporting environmental waste management.

## 1. Introduction

In recent years, natural fiber–reinforced polymer composites (NFPCs) have increasingly begun to replace conventional materials in both industrial applications and academic research, driven by concerns over environmental sustainability and the deamand for lightweight materials Aranguren et al. [1]. Composite materials are heterogeneous structures formed by combining a matrix phase with a reinforcing phase, thereby offering superior properties that the individual constituents cannot provide on their own Saindane et al. [2]. Particularly in the automotive sector, the need to reduce weight and comply with emission criteria has led researchers to seek biodegradable and renewable resources as alternatives to synthetic fibers Şimşir et al. [3]. In this context, utilizing agricultural and animal-derived wastes in composite production not only addresses waste disposal problems but also enables the development of low cost, environmentally friendly materials Ertürk and Kıratlı [4]. The advantages of natural fibers over synthetic fibers such as low density, low cost, and acceptable specific strength have rapidly increased the use of these materials across various sectors including construction, automotive, and packaging Keya et al. [5]. Moreover, employing animal-based wastes as reinforcement elements in polymer composites has emerged as an important strategy that supports economic and socially sustainable development [6,7].

Among natural reinforcement materials, in addition to plant-based fibers, keratin-based animal wastes particularly structures such as horns and hooves have attracted considerable attention due to their outstanding mechanical potential Huang et al. [8]. Goat horn powder is composed of α-keratin, one of the most durable biological materials found in nature, and it plays a critical role in animals’ defense mechanisms Zhang et al. [9]. Unlike mineralized tissues such as bone or teeth, these keratin-based materials exhibit high toughness, hardness, and impact resistance that help prevent brittle fracture [10,11]. Studies have shown that horn structures possess the ability to dissipate energy even under high-velocity impacts, and that this behavior is closely associated with moisture content [12,13]. For instance, the horns of bighorn sheep and other members of the Caprinae subfamily have evolved to protect the skull by absorbing impacts, displaying a hierarchical architecture consisting of a porous bony core and a keratin sheath Yang et al., Kumar and Boopathy [14,15]. Investigations on the horns of local breeds, such as the India-origin Deccani sheep, have further indicated that these materials may serve as alternative biomaterials for structural applications owing to their high compressive strength and favorable specific gravity [16,17]. Therefore, converting horns generated as slaughterhouse waste into powder for use in composites not only contributes to waste management but also offers a lightweight and durable alternative to synthetic fillers Laynde et al. [18].

To maximize the potential of such keratin-based natural reinforcements, selecting a polymer matrix that is compatible with their chemical structure and can be shaped into the desired forms is of critical importance. Polyurethane (PU), owing to its versatile chemistry and superior mechanical properties, is widely preferred as a matrix material, particularly for producing wood-like structures and foams [19,20]. Polyurethanes, which can be manufactured in either thermoset or thermoplastic forms, are capable of forming chemical bonds between isocyanate groups and hydroxyl groups present on the surface of natural fillers, thereby strengthening interfacial adhesion Fornasieri et al. [21]. Recent studies have shown that bio-based polyurethane wood composites (PU-WCs) produced using biomass-derived polyols illustrate how interphase interactions govern overall material performance Olszewski et al. [22]. Experiments on polyurethane composites reinforced with biological wastes such as cattle hoof powder have demonstrated that, at certain loading levels, reinforcement can increase tensile strength and modulus while also modifying physical properties such as water resistance. When polyurethane’s low-density yet high-strength structure is combined with wastes such as horn powder, it enables the development of next-generation composites that are sustainable from both economic and environmental perspectives Nathan et al. [23], Chalivendra et al. [24].

To verify the industrial applicability of these newly developed composites, a comprehensive mechanical analysis process is required. While the strength of a material is assessed by its ability to withstand applied stress, parameters such as hardness, toughness, and impact resistance determine its performance under dynamic loading conditions Ateş [25]. Studies in the literature indicate that particle size and reinforcement content (wt.%) have a direct influence on the tensile, flexural, and impact performance of composites. For example, in aluminum alloys reinforced with cow horn particles, a marked increase in hardness has been observed with increasing particle fraction, whereas decreases in absorbed impact energy have been reported Joshua et al. [26]. Similarly, tests on animal-fiber-reinforced polymers have shown that increasing fiber content improves the tensile modulus; however, once the optimum level is exceeded, mechanical properties deteriorate due to matrix–fiber incompatibility Antor et al. [27]. In polyurethane composites reinforced with cattle hoof powder, tensile strength increased up to a 7.5 wt.% loading, but at higher contents performance declined because the filler restricted matrix mobility and agglomeration became more pronounced. Therefore, systematic application of hardness (Shore D or Rockwell), tensile, and impact tests is essential for understanding the mechanical behavior of the produced composites Osoka et al. [28].

Microstructural investigations are indispensable for fully understanding the origin of macroscopic data obtained from mechanical tests and for optimizing material design. Scanning Electron Microscopy (SEM) analyses reveal, in detail, the dispersion of reinforcement phases within the matrix, the quality of interfacial adhesion, and fracture mechanisms (e.g., fiber pull-out, matrix cracking, etc.) Ceritbinmez et al. [29]. It is well known that tubular and lamellar arrangements in horn structures play a critical role in energy dissipation and in mechanisms that arrest crack propagation Tombolato et al. [10]. For instance, microscopic examinations of fracture surfaces after impact testing have confirmed that moist horn structures absorb energy by exhibiting plastic deformation, whereas dry structures tend to show brittle fracture behavior Huang et al. [30]. In addition, SEM analyses of epoxy composites reinforced with ammonium polyphosphate and goat horn have demonstrated that homogeneous filler dispersion directly affects mechanical performance, while agglomerations create weak regions Huang et al. [8]. Studies on the microstructure of Caprinae horn sheaths have further revealed that wavy interfaces and hierarchical microfibers trigger complex fracture mechanisms, such as delamination and crack deflection Baba and Baba [31].

The aim of this study is to systematically investigate the hardness, microstructure, and mechanical properties of Honamlı goat horn powder–reinforced wood-like polyurethane composites. The composites were fabricated at different horn powder concentrations (5–25 wt.%), and their microstructural interfaces were examined using SEM images. Mechanical performance was evaluated through Shore D hardness testing, three-point bending tests, and tensile tests. The findings contribute to the development of next-generation, sustainable, high-performance polyurethane composites derived from biological waste and demonstrate the potential of these materials for decorative, lightweight structural, and impact-absorbing applications.

## 2. Materials and Methods

### 2.1. Materials

**Figure 1:**
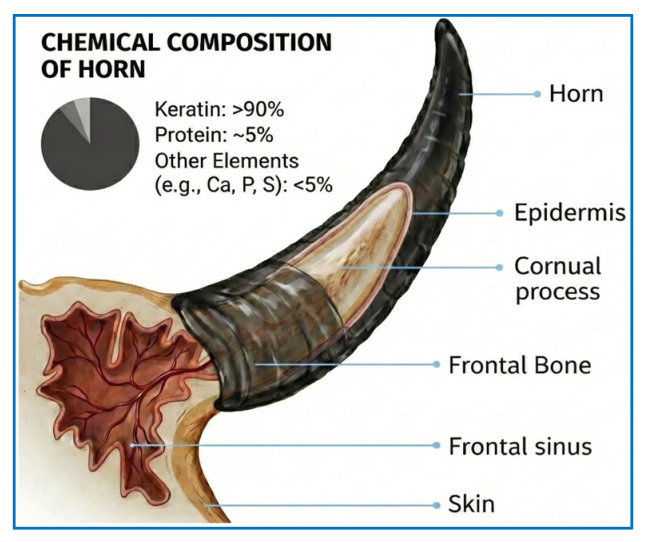
Internal structure of the goat horn.

Horn is generally regarded as an extension of a bony structure in mammals, located on the anterior part of the skull, with a conical cross-section and a curved or spiral form. This extension, which forms through thickening and morphological alteration of the epidermal tissue, is composed of keratin, other proteins, and elements that cover its outer surface Taşkın et al. [32]. An illustration of the internal structure of the horn, which exhibits resistance to both mechanical and chemical effects, is presented in Figure 1.

In Table 1, a table showing the main (predominant by weight) elements and trace elements that make up the general chemical structure of GHP is provided Sule et al. [33].

In this study, the mechanical and chemical properties of the materials used in the preparation of the goat horn powder reinforced polymer composite are given in Table 2 below.

The values reported in Table 2 are literature data for the natural goat horn structure (solid keratin). The relatively high ultimate tensile strength (UTS) of 95 MPa arises from the horn’s hierarchical, lamellar solid keratin architecture. The low density, on the other hand, is a consequence of the horn’s naturally porous and lightweight biological design. Although the grinding process eliminates this macroscopic strength, the table is included to present the material’s chemical/natural characteristics and to provide a contextual baseline for the reinforcement used in this study.

**Figure 2:**
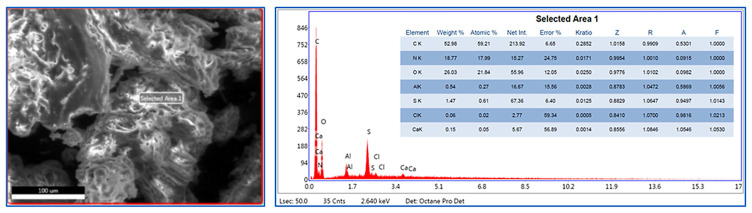
EDS analysis of goat horn powder.

Below, an EDS image of pure GHP (at a 100 µm scale) is presented in Figure 2. Here, it is observed that the powder particles have irregular geometries, a rough surface structure, and a locally layered texture. This morphology represents the typical fracture characteristic exhibited by animal (keratin-based) tissues after a mechanical grinding process. The surface roughness is considered to provide an advantage for mechanical interlocking if this powder is used as a reinforcing element within a polymer matrix (e.g., polyurethane). The analysis results prove that the sample has a keratin structure, which is a natural biopolymer strengthened by sulfur bonds and characterized by high protein content. In particular, the high nitrogen and sulfur contents indicate that this material is distinct from plant-derived carbohydrate structures and can be stated to exhibit the characteristics of pure animal keratin.

### 2.2. Preparation of Goat Horn Powder (GHP)

In this study, the goat horn used as the reinforcing element was obtained from the Honamlı goat, which is raised as a local breed in the geographic region of Türkiye commonly referred to as the “Teke region.” Goat horn powder was selected because Honamlı goats are widely raised in the Burdur/Teke region, enabling easy local procurement and a traceable supply chain. The horns constitute a local by-product/waste stream, and their valorization aligns with our sustainability objective while reducing transportation-related impacts. Although other keratin-based wastes (e.g., cattle or deer) may also be considered, they were not included here due to limited regional availability and the additional variability they could introduce in supply and logistics; a comparative assessment of different keratin sources is planned for future work.

**Figure 3:**
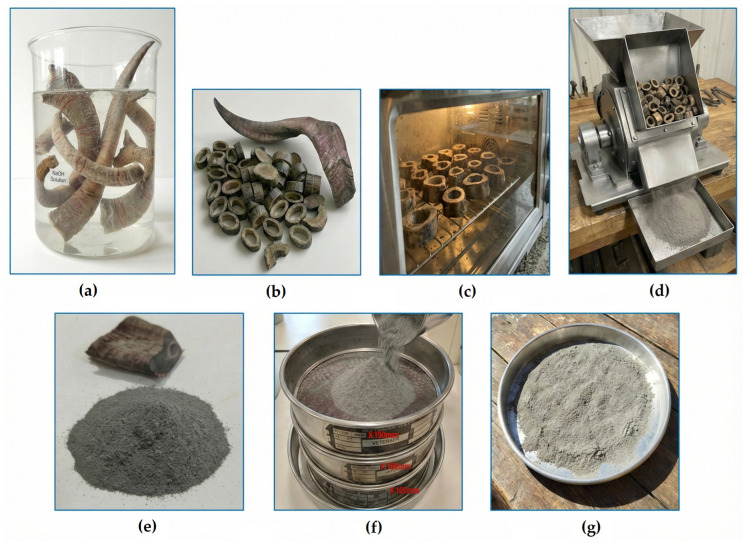
GHP preparation stages: (**a**) Cleaning of goat horns in NaOH solution, (**b**) cutting into small pieces, (**c**) moisture removal in an oven, (**d**) grinding the horn into powder in a grinder, (**e**) separation of the powders from metals, (**f**) sieving process, (**g**) drying of the horn powders.

The horns were supplied from a local slaughterhouse where slaughtering operations are carried out to meet meat consumption demands. The horns, which are considered animal waste, were first cleaned with abundant water using a brush to remove hard and foreign substances. Then, to eliminate impurities, they were kept in a sodium hydroxide (NaOH) solution for 24 h. Afterwards, they were immersed in distilled water for 1 h to remove the chemical solution. The horns were then left to dry in open air for 3 days. Subsequently, they were cut into small ring-shaped pieces using a saw, with the aim of ensuring faster and more uniform drying. Following these procedures, the horn was dried in an oven at 60 °C for 2 h, and after being removed from the oven, it was fragmented using a mixer–grinder until it became a fine powder. After this process, metal pieces were collected with the aid of a magnet to eliminate metal particles that could mix into the powders. The horn powders were left to dry again at atmospheric temperature for 48 h to completely remove moisture. The dried goat horn powders (GHP) were sieved in a vibratory sieve device with particle mesh sizes of 150 µm, 125 µm, and 100 µm, thereby making them dimensionally suitable for composite production. The process steps of all these procedures are shown in Figure 3.

### 2.3. Specimen Preparation of GHP Reinforced Wood-like Polyurethane Composites

#### 2.3.1. Polyurethane Matrix Characteristics

The binder system used in this study is a two-component commercial polyurethane resin. Component A is a polyol-based resin phase containing filler, whereas Component B is an isocyanate-based hardener. According to the manufacturer’s technical data sheet, the system is formulated with a polyol/isocyanate mixing ratio of 100/100 by weight. This ratio indicates an approximately stoichiometric balance between hydroxyl (–OH) and isocyanate (–NCO) functional groups. Polyurethane formation is based on an addition reaction between the hydroxyl groups of the polyol and the isocyanate groups of the isocyanate component (Equation (1)):

R–OH + R′–NCO → R–NH–CO–O–R(1)

As a result of this reaction, urethane (carbamate) linkages are formed, and in the presence of multifunctional reactants, a three-dimensional crosslinked polymer network develops. The free NCO content reported for Component B in the range of 21.3–22.2% indicates that the system has high reactivity and a significant potential for crosslinking. In the literature, such an NCO range is generally associated with MDI-type isocyanate-based systems and suggests the formation of rigid/semi-rigid polyurethane network structures.

During curing, it is known that isocyanate groups can react not only with the polyol but also with trace amounts of water present in the environment (Equation (2)):

R–NCO + H_2_O → R–NH_2_ + CO_2_ (2)

The amine groups formed can subsequently react with another isocyanate group to form urea linkages. These side reactions may also increase the stiffness and crosslink density of the network. Therefore, the system may form a hybrid poly (urethane–urea) network containing not only urethane bonds but also a certain fraction of urea segments.

In order to manufacture the test specimens, the production of the positive model and the negative mold was carried out sequentially. The positive models were produced in three different standards using a three-dimensional printer with the Fused Deposition Modeling (FDM) method. In determining these standards, standard geometries applied for plastic materials were selected by taking into account similar studies and recommendations in the literature. Accordingly, for tensile, impact, and three-point bending tests, specimen geometries were determined in accordance with the standards of the American Society for Testing and Materials (ASTM), namely ASTM D638 [39], ASTM D256 [40], and ASTM D790 [41], respectively. All standard specimen geometries were manufactured on a Bambu LAB P1S 3D printer using the specified production parameters and ABS (Acrylonitrile Butadiene Styrene) material. As FDM machine parameters, a 0.4 mm nozzle diameter and a 0.2 mm layer thickness were selected. The specimens were printed with 100% infill using a linear infill pattern, and the printing orientation was set parallel to the loading axis. While the nozzle temperature was maintained at approximately 240 °C, the build plate temperature was set in the range of 90–100 °C. The printing speed and cooling settings were optimized to enhance interlayer adhesion and ensure dimensional stability. The standard specimens produced at this stage were used as negative molds for the composite casting processes.

**Figure 4:**
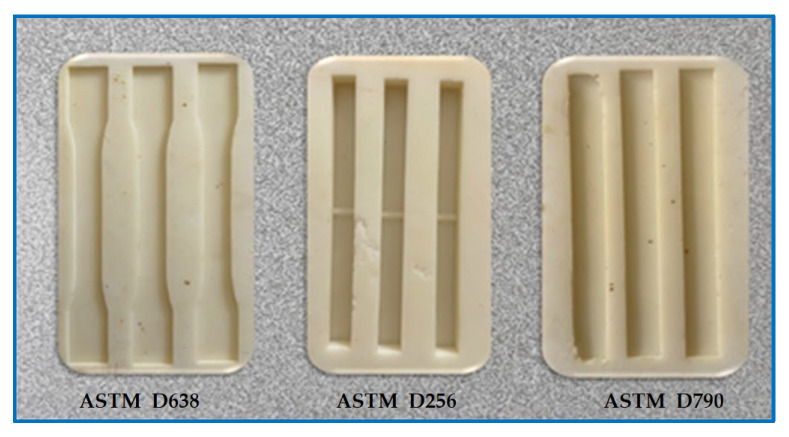
Composite material production molds, in order; tensile test, charpy impact, 3-point bending.

To prepare the negative molds, the positive models were placed inside the mold containers. Care was taken to maintain the axial alignment of the molds and to preserve the integrity of the standard specimen geometry. The two-component silicone rubber selected as the mold material (AK-SİL S-Series RTV-2) was poured into the containers slowly and in a controlled manner to reduce air entrapment and bubble formation, thereby forming the negative molds. After casting, the silicone molds were allowed to cure at room temperature for 24 h. The curing time was determined according to the manufacturer’s specifications to ensure sufficient mechanical strength of the mold. After the curing process, the models were gently removed from the negative molds, and in accordance with ASTM requirements, the accuracy of the internal cavity geometry was verified by visual inspection and dimensional validation using a caliper, as shown in Figure 4.

#### 2.3.2. Preparation Process of the Mold

**Figure 5:**
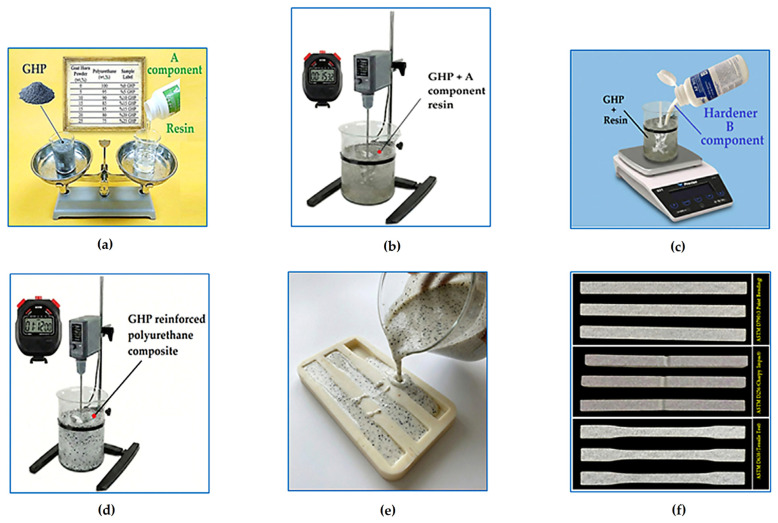
Preparation stages of GHP-reinforced composites: (**a**) calculation of the mixture weights of GHP and resin, (**b**) mixing of the prepared composition, (**c**) addition of the hardener to the mixture, (**d**) mixing of all components, (**e**) casting the composite into the mold, (**f**) demolding of the specimens.

The composite specimens were produced using goat horn powder (GHP) and a two-component wood-like polyurethane system. The powders with an approximate size of 100 µm obtained from the powder preparation process whose stages are given in Figure 3 were weighed according to the reinforcement levels determined in the experimental design (5 wt.%, 10 wt.%, 15 wt.%, 20 wt.%, and 25 wt.%). The weighed powders were then incorporated into the casting process, respectively. An important point here is that a mixer was used to ensure that the powder and the A component of the polyurethane could be mixed homogeneously. Imanullah et al. [42] conducted microstructural observations to investigate the homogeneous distribution of particles. In this context, the distance of the mixer from the bottom of the container was tested at three different levels corresponding to 30%, 40%, and 50% of the liquid height. Microstructural analyses showed that the best homogeneous distribution was obtained in mixtures where the mixer was positioned at 40% of the liquid height from the bottom. Therefore, the same mixer height (40%) was used in the preparation of composites containing goat horn powder. The preparation stages of the goat horn powder–reinforced wood-like polymer composite are shown in Figure 5. The mixture of GHP and the A component of the ANCAPOL AP902 wood-like polyurethane resin was prepared by mixing for 1 h using a mechanical mixer. Then, the B component (hardener) was added to the mixture and rapidly mixed. The resulting new mixture was poured into the molds within 72 s, known as the critical time interval, and the composite specimen production process was completed without any errors. The low viscosity of 100–200 mPa·s at 25 °C allows efficient mixing of the reactive species and good wetting of the substrate/reinforcement surfaces. A pot life of approximately 130–140 s indicates fast reaction kinetics and rapid gel formation. The tack-free (touch-dry) time of 300–360 s further supports the rapid development of the three-dimensional network. These data confirm that the system exhibits a typical thermoset polyurethane character. The fact that the specific gravities of both components fall within a similar range (1.22–1.25 g/cm^3^) helps maintain homogeneity during mixing and reduces the likelihood of phase separation. This is important for the structural continuity of the resulting polymer matrix. In conclusion, the chemical class of the system (two-component polyurethane), the reaction mechanism (–OH/–NCO addition), the reactivity level (NCO percentage), and the curing kinetics have been sufficiently defined on the basis of the manufacturer’s data. Since the scope of the study is the investigation of macroscopic mechanical and/or performance behavior, the specific polyol molecular weight or the detailed commercial chemical name of the isocyanate in the commercial formulation does not limit the scientific interpretation of the results. The composite poured into the molds was left to solidify at room temperature for 24 h. At the end of this period, the specimens were carefully removed from the mold. Throughout all these stages, care was taken to ensure a homogeneous powder distribution within the matrix, thereby guaranteeing the structural integrity of the composites. Similarly, the mixing and casting process was repeated for all specimens by considering the differences in weight ratios in the production stages. Upon completion of the composite production process, three specimens were produced for each test standard.

The composite preparation process, whose production stages are schematically presented above in Figure 5, was repeated for each specimen according to the weight ratios determined. Along with the neat polyurethane specimen, the table of the composite materials produced with five different weight compositions is given below in Table 3.

### 2.4. Methods

#### Mechanical Testing of GHP Reinforced Wood-like Polyurethane Composites

**Figure 6:**
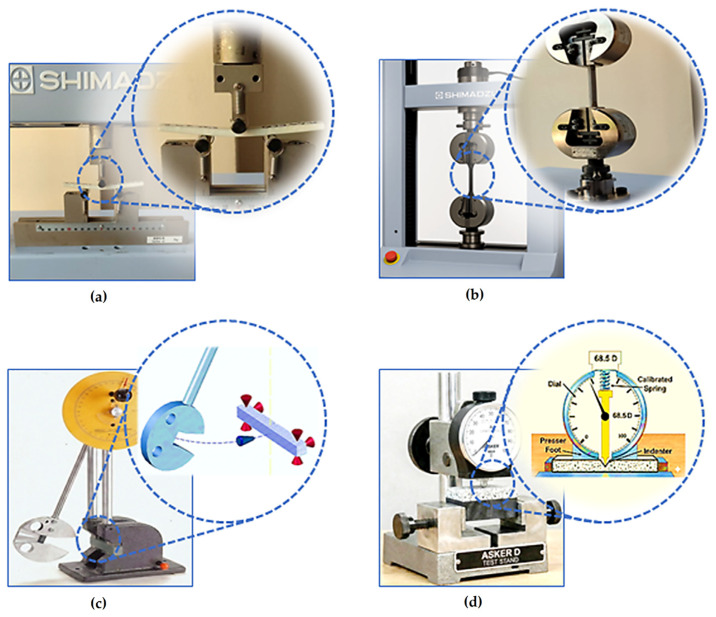
Mechanical testing devices: (**a**) three-point bending test, (**b**) tensile test, (**c**) charpy impact test, (**d**) shore D hardness test.

To evaluate the mechanical behavior of the produced goat horn powder–reinforced polyurethane composites, a comprehensive mechanical characterization program was carried out, including tensile, three-point bending; Charpy notched impact, and Shore D hardness tests. All test specimens were prepared in accordance with the relevant polymer testing standards ASTM D638 (tensile), ASTM D790 (flexural), and ASTM D256 (Charpy notched impact) to ensure comparability and consistency between measurements. Tensile and three-point bending tests were performed on a Shimadzu AGS-X (Shimadzu Corporation, Kyoto, Japan) electromechanical universal testing machine equipped with a 10 kN load cell. The machine operates with a crosshead speed range of 0.001–1000 mm/min and provides data with 0.1% accuracy. The Charpy impact strength (IS) of the composites produced in five different compositions was measured using an impact testing device (MT-3016, pendulum type). All results were taken as the average values of three specimens. Shore D hardness measurements were conducted using an ASKER Shore D durometer. The hardness measurements were performed in accordance with ASTM D2240 [43]. Prior to testing, specimen surfaces were prepared to be flat and parallel, and the specimen thickness was prepared according to the ≥6 mm requirement specified for this standard. During hardness measurements, indentation points were selected at least 12 mm from the specimen edges, and a minimum distance of 6 mm was left between two measurement points. The durometer indenter was applied perpendicularly to the surface in a smooth manner to prevent impact loading and any lateral force. After full contact of the presser foot was achieved, readings were recorded following a waiting time of at least 1 s. For each specimen, three measurements were taken at different locations, and the reported hardness value was calculated as the average of these readings. A schematic summary of the application of all these mechanical tests is presented below in Figure 6.

The surface morphology, microstructural characteristics, and elemental composition of the samples were examined using an FEI Quanta FEG 250 field-emission scanning electron microscope (FE-SEM) (Thermo Fisher Scientific, Osaka, Japan) located at the Innovative Technologies Application and Research Center (YETEM) Laboratory, Süleyman Demirel University. The analyses were carried out at an accelerating voltage of 20 kV using a Large Field Detector, under a chamber pressure of 70 Pa, at a magnification of 1000×. Thanks to the microscope’s high-resolution field emission gun (FEG) source, high-resolution images were obtained even at low voltages. Prior to the analyses, the specimens were appropriately cut and, when necessary, their surfaces were flattened by polishing in order to minimize the effect of surface roughness on image quality. SEM examinations were performed at different magnifications to reveal the specimen surface morphology and the matrix–reinforcement relationship. In addition, qualitative and quantitative elemental analyses of the specimens were conducted using an EDS detector integrated into the SEM instrument (Thermo Fisher Scientific, Waltham, MA, USA). Both the elemental distribution and the interfacial adhesion between the goat horn powder and the wood-like polymer matrix, as well as possible agglomeration behavior, were evaluated. The distribution density of the reinforcement within the matrix, together with the reinforcement–matrix interaction, and the effects of crack formation, micro-voids, and similar internal structural defects on composite performance were investigated.

## 3. Results and Discussion

### 3.1. Mechanical Testing of GHP Reinforced Polyurethane Composites

#### 3.1.1. Tensile Test Results

**Figure 7:**
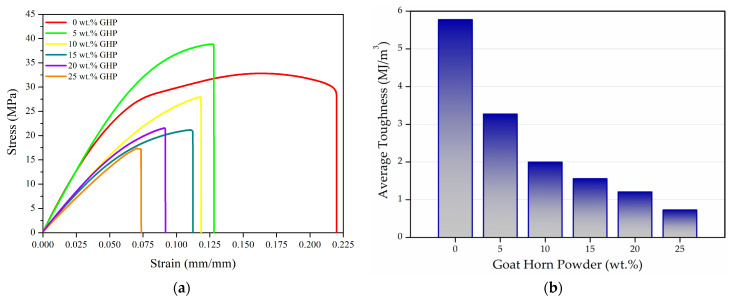

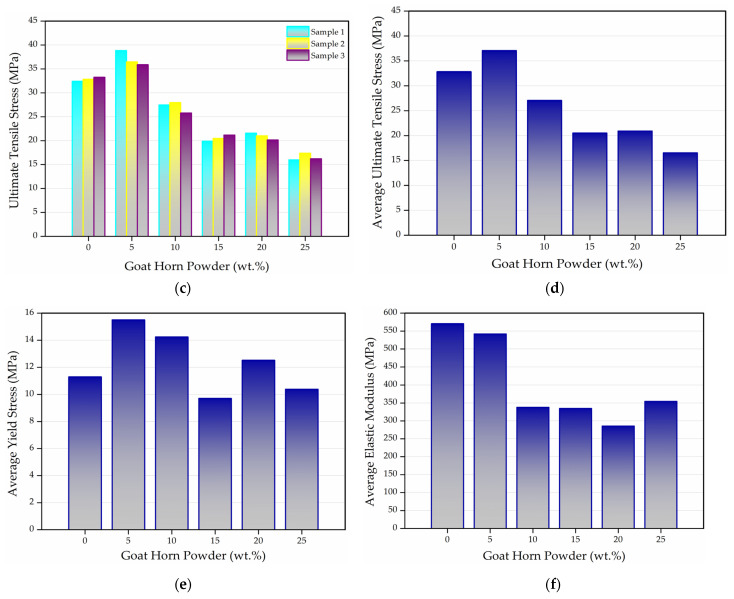
Graphs of the tensile test data of the composites: (**a**) stress–strain, (**b**) average toughness, (**c**) maximum tensile stress of all specimens, (**d**) average maximum tensile stress, (**e**) average yield stress, and (**f**) average elastic modulus.

To evaluate the tensile behavior of wood-mimicking polyurethane matrix composites containing goat powder (GHP), five different formulations with GHP contents ranging from 5 to 25 wt.% were prepared. For each formulation, three specimens were produced under identical manufacturing conditions, and the tensile test results obtained from these specimens are presented graphically in Figure 7.

When Figure 7a,c,d are evaluated together, it is observed that the material exhibits increasingly brittle behavior with increasing GHP content, accompanied by a marked reduction in elongation at break. This trend is thought to arise from factors such as non-uniform dispersion and agglomeration of the reinforcement in certain regions of the microstructure, insufficient interfacial adhesion between the filler and the matrix, and a reduced ability of the polymer matrix to form a continuous chain network. Otto, et al. reported that high reinforcement contents led to agglomeration and interfacial defects, which increased stress concentration and crack initiation propensity, ultimately resulting in brittle fracture [44]. In contrast, the addition of 5 wt.% reinforcement was found to increase the average maximum tensile stress of the composite by 12.89%; however, at higher reinforcement contents, the average maximum tensile stress decreased compared to that of the neat matrix. This suggests that reinforcement up to 5 wt.% provides resistance by hindering crack propagation within the microstructure, whereas higher loadings may accelerate crack formation, leading to a reduction in the average maximum tensile stress. Nunes et al. reported that at low filler contents, filler particles interact with growing cracks and enhance viscoelastic energy dissipation, thereby delaying crack propagation and increasing tensile strength; conversely, at high filler contents, the formation of voids at the filler–matrix interface and the resulting increase in stress concentrations reduce tensile strength [45]. In another study, Atagur et al. reported that the increase in stress observed at low reinforcement contents was due to the dispersed reinforcement within the matrix restricting the elastic chain mobility of the polymer, thereby providing higher resistance against tensile loading [46]. In other words, the rise in tensile stress at low reinforcement levels can also be explained by a more homogeneous distribution of GHP particles within the matrix, contributing to load transfer through mechanical interlocking. This “reinforcement threshold” behavior observed in natural fiber/filler-reinforced polymers at low reinforcement contents has been widely reported in the literature as an increase in tensile strength at reinforcement levels where matrix–reinforcement interactions remain sufficiently effective [47]. Serrano Martínez et al. reached similar conclusions in their study published in 2024, stating that keratin-based natural fillers (e.g., horn, feather, nail, etc.) can exhibit a reinforcing effect at low loadings; however, once a critical content is exceeded, mechanical performance begins to deteriorate due to the disruption of matrix continuity [48].

Figure 7b presents the toughness values calculated by determining the area under the stress–strain curve. A decrease in the toughness of the composite was observed as the amount of reinforcement added to the matrix increased. With the addition of 5 wt.% GHP, the toughness of the composite decreased by 43.85% compared to neat polyurethane, whereas increasing the reinforcement content to 25 wt.% resulted in an 87.39% reduction in toughness. Although a slight increase in maximum tensile stress is obtained at 5 wt.% GHP, the stress–strain curves indicate a pronounced reduction in elongation at break and a narrowing of the plastic deformation region, i.e., reduced ductility. Since toughness is defined here as the area under the stress–strain curve, the loss in strain (energy absorption capacity) outweighs the modest strength gain, resulting in a net decrease in toughness. This behavior is consistent with rigid, discontinuous particles promoting local stress concentrations and triggering earlier damage mechanisms such as microvoid initiation and particle–matrix interfacial debonding when interfacial adhesion is not fully optimized.

The increasingly brittle behavior exhibited by the composite with higher reinforcement contents appears to be the primary reason for the decline in toughness. In natural reinforcement-based composites, the reduction in deformability and the rise in brittle fracture with increasing reinforcement content are frequently emphasized in the literature. In particular, as the reinforcement content increases, the distance between reinforcement particles decreases, preventing the matrix from fully encapsulating the reinforcement and promoting interfacial void formation, this ultimately leads to premature failure [49].

When Figure 7e,f are evaluated together, the average yield stresses of the composites produced with 5, 10, and 20 wt.% reinforcement contents were measured to be higher than that of the unreinforced polyurethane. However, as the amount of reinforcement increased, a decrease in the average elastic modulus of the composites was observed. In addition to embrittling the polymer, the incorporation of reinforcement into the matrix may alter the reaction kinetics, crosslink density, or microstructure of the polymer; therefore, it is considered that certain reinforcement levels may lead to an increase in yield stress, while a reduction in elastic modulus occurs at all reinforcement contents. The literature similarly reports that the use of bio-based reinforcements in polyurethane-based composites can increase structural heterogeneity and void formation, which may simultaneously reduce both modulus and strength in some polymer matrix composites [50]. On the other hand, it is also known that in some polymer matrix composites reinforced with lignocellulosic fillers, stiffness can increase at appropriate reinforcement levels [51].

#### 3.1.2. 3 Point Bending Test Results

**Figure 8:**
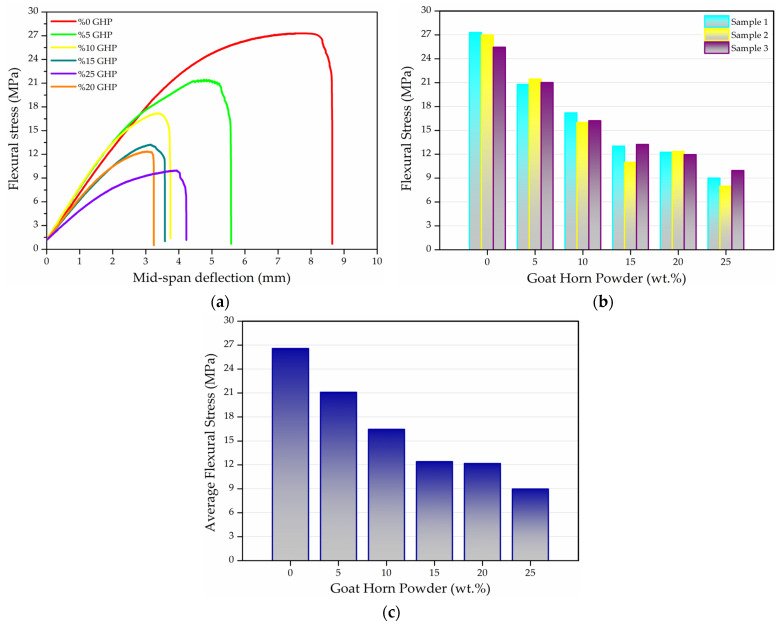
Graphs of the three-point bending test: (**a**) Flexural stress-mid. span deflection, (**b**) Flexural stress of all specimens, (**c**) Average flexural stress.

To evaluate the three-point bending behavior of wood-mimicking polyurethane matrix composites containing goat powder (GHP), five different formulations were prepared with GHP contents ranging from 5 wt.% to 25 wt.%. For each formulation, three specimens were produced under identical manufacturing conditions, and the data obtained from the three-point bending tests applied to these specimens are presented in Figure 8a–c in the form of graphs.

When Figure 8a,b are evaluated together, the maximum flexural stress of the unreinforced polymer was measured as 26.59 MPa, while the maximum mid-span deflection reached 8.64 mm. Among the produced composites, the highest flexural stress was obtained for the 5 wt.% reinforced composite, with a value of 21.08 MPa. The lowest deflection occurred in the 20 wt.% reinforced composite, with a value of 3.24 mm. Compared to the neat polymer, the flexural stress of the 25 wt.% reinforced composite decreased by 66.23%. The maximum reduction in deflection was observed in the 20 wt.% reinforced composite, showing a decrease of 62.5%.

The systematic decrease in flexural strength observed with the addition of GHP can be attributed to the increased tendency of bio-based particles to agglomerate as the filler content rises, as well as the inability to achieve effective load transfer due to weak reinforcement–matrix interfacial adhesion and the formation of microvoids. In the studies conducted by Fu et al. and Lee et al., it was reported that stress concentrations formed around the reinforcement particles act as early damage initiation sites, leading to a more brittle fracture behavior under flexural loading [52,53]. In the studies published by Członka et al. and Aranberri et al., it was demonstrated that bio-based reinforcements exhibit weak interfacial adhesion within a polyurethane matrix, thereby limiting effective load transfer. As a result, microcrack formation was accelerated, leading to reductions in both flexural strength and deflection capacity [54,55]. Similarly, the works of Talabi et al. and Duraisamy et al. reported that the mechanical performance of polyurethane composites reinforced with animal-derived particulate fillers varies markedly depending on the reinforcement content, and that at high filler loadings, the particles may behave more as defect sources rather than effective reinforcing agents [56,57].

#### 3.1.3. Charpy Impact Test Results

Charpy impact tests were conducted to evaluate the impact behavior of composites produced by incorporating goat horn powder into a neat wood-like polyurethane matrix at weight fractions of 5 wt.%, 10 wt.%, 15 wt.%, 20 wt.%, and 25 wt.%. For comparative assessment, unreinforced neat polyurethane specimens were also fabricated as the control group. A total of 18 specimens were tested, with three specimens prepared for each composition and the control group. This approach enabled both the reproducibility of the measurements and the reliable identification of changes in impact energy as a function of reinforcement content. In this context, the first graph presents the Charpy impact energy values obtained from three individual specimens for each test set, illustrating the specimen-to-specimen variation, whereas the second graph shows the arithmetic mean of the three measurements to more clearly reveal the overall trend in the impact strength of the polyurethane matrix with the addition of goat horn powder.

**Figure 9:**
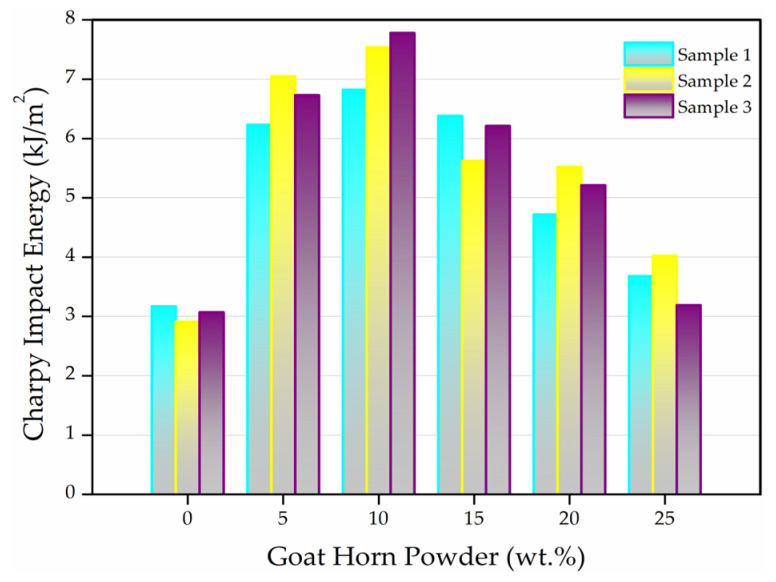
Charpy impact energy values of all specimens.

The Charpy impact test results in Figure 9 indicate that the incorporation of goat horn powder (GHP) into the wood-like polyurethane (PU) matrix improves the impact energy absorption behavior up to certain reinforcement levels, whereas this beneficial effect diminishes at higher filler contents. The neat PU control specimen exhibited an average Charpy impact energy of 3.05 kJ/m^2^. With the addition of 5 wt.% GHP, the impact energy increased to 6.67 kJ/m^2^, corresponding to an improvement of approximately 118% compared to neat PU. The highest impact energy was achieved at 10 wt.% GHP, reaching 7.38 kJ/m^2^, which represents an enhancement of about 140% relative to the control. However, when the GHP content exceeded 10 wt.%, a gradual decrease in impact energy was observed, and at 25 wt.% GHP the average impact energy dropped to 3.63 kJ/m^2^, approaching the value of neat PU. The consistency of the impact energy values obtained from the three replicate specimens for each composition demonstrates good experimental repeatability. Moreover, the pronounced differences observed between low (5–10 wt.%) and high (≥15 wt.%) GHP contents suggest that the variations in impact performance are likely statistically meaningful rather than attributable to experimental scatter.

The Charpy impact results demonstrate that goat horn powder acts as an effective toughening agent for the wood-like PU matrix at low filler loadings; however, excessive filler addition leads to deterioration in impact performance. The pronounced improvement observed within the 5–10 wt.% GHP range can be attributed to energy-dissipation mechanisms activated during impact loading. At these filler levels, GHP particles are presumed to be relatively homogeneously distributed within the PU matrix, promoting mechanisms such as crack deflection, micro-crack formation, and increased energy consumption associated with enhanced friction at the particle–matrix interface. Collectively, these mechanisms delay crack propagation, enabling greater energy absorption under impact conditions and thereby increasing the overall toughness of the composite.

This behavior is consistent with previous studies on natural filler and fiber-reinforced polyurethane systems, where improvements in impact strength and fracture toughness have been reported at optimum reinforcement levels. For instance, Lopes et al. reported a pronounced increase in Charpy impact performance in polyurethane composites reinforced with bamboo waste at intermediate reinforcement contents, attributed to improved stress transfer and enhanced energy absorption capacity [58]. Similarly, Bakare et al. demonstrated that low-to-moderate reinforcement levels in sisal fiber reinforced biobased polyurethane composites promote effective load sharing, leading to significant improvements in impact resistance [59].

In contrast, the gradual decrease in impact energy observed when the GHP content exceeds 10 wt.% can be associated with disruption of the continuous PU matrix phase and the increased tendency of particles to agglomerate at high filler loadings. Such agglomerates act as stress concentration sites around the particles, facilitating premature crack initiation and thereby reducing the impact energy absorption capability. Indeed, El-Shekeil et al. reported a marked reduction in impact strength with increasing fiber content in kenaf fiber–reinforced TPU composites, attributing this behavior to poor dispersion and intensified fiber–fiber interactions at higher reinforcement levels [60]. In a similar manner, studies by Sair et al. and Kumar and Boopathy indicated that, in polymer composites reinforced with natural fibers or horn/keratin-based biofillers, mechanical performance deteriorates beyond an optimum reinforcement level due to increased defect density and a greater tendency toward brittle fracture [15,61]. Moreover, Parkunam et al. emphasized that when goat horn based reinforcements are used at high concentrations, the particles may behave as defect sources rather than effective reinforcement elements, thereby limiting impact performance [13].

When the results of the three replicate specimens for each composition are examined in Figure 9, the impact energy values at 5 and 10 wt.% GHP are seen to be highly consistent, whereas the scatter between replicates increases at higher filler contents. This trend can be attributed to reduced dispersion homogeneity during processing and an increased sensitivity to localized agglomeration as the GHP loading rises. Overall, the Charpy impact tests clearly demonstrate that goat horn powder exhibits an optimum filler range for enhancing impact toughness in PU-based composites, with the most balanced and highest impact performance achieved at 10 wt.% GHP in the present study.

#### 3.1.4. Shore D Hardness Test Results

**Figure 10:**
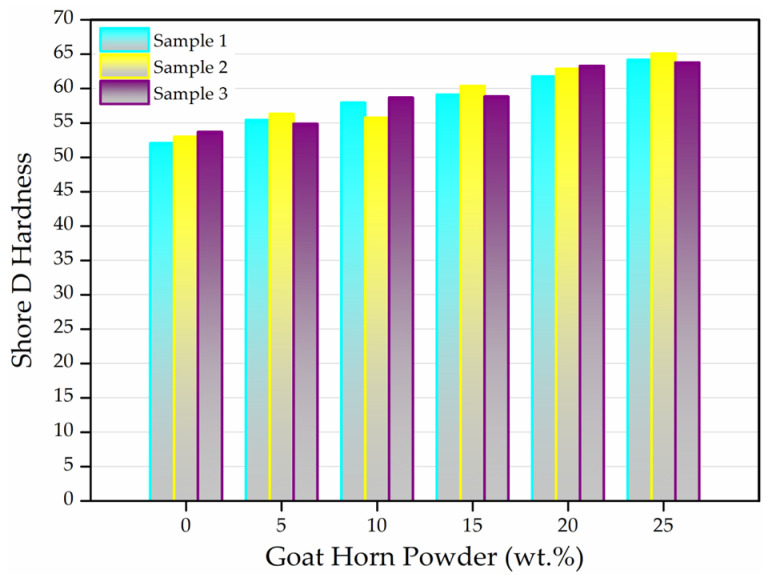
Shore D hardness values of all specimens.

The surface hardness of the composites produced by reinforcing the wood-like polyurethane matrix with different contents (5–25 wt.%) of goat horn powder (GHP) was evaluated using Shore D hardness tests. Unreinforced neat polyurethane specimens were used as the control group, and three replicate measurements were performed for each composition to assess the consistency of the results. Figure 10 presents the individual hardness values corresponding to each reinforcement level, thereby revealing the overall effect of GHP reinforcement on hardness.

The Shore D hardness test results indicate that goat horn powder (GHP) increases the surface hardness of the wood-like polyurethane (PU) matrix in a regular and systematic manner as a function of reinforcement content. While the hardness values of the neat PU specimens were measured to be approximately within the range of 52–54 Shore D, a noticeable increase was observed with the incorporation of 5 and 10 wt.% GHP, and this upward trend continued at 15, 20, and 25 wt.% GHP loadings. The highest hardness values were obtained at 20 and 25 wt.% GHP, demonstrating that GHP acts as an effective stiffening reinforcement phase within the PU matrix.

This increasing trend can be attributed to the inherently high stiffness and hardness of the hierarchical microstructure of α-keratin, which is the main constituent of goat horn. Tombolato et al. reported that horn keratin exhibits high resistance to local deformation due to its lamellar and fibrous architecture [10]. Likewise, Lazarus et al. demonstrated that keratin-based structures can behave as a stiffening reinforcement phase in polymer systems and enhance surface hardness [62]. In particulate-reinforced polyurethane composites, hardness improvement is commonly explained by the restriction of polymer chain mobility caused by the reinforcing particles and the resulting increase in local stiffness under indenter loading. In their comprehensive study, Fu et al. emphasized that reinforcement–matrix interfacial interactions and increasing filler content directly influence stiffness-controlled mechanical properties such as hardness [52]. Similarly, Bakare et al. reported a systematic increase in hardness and stiffness with increasing reinforcement content in natural fiber–reinforced polyurethane composites [59]. Haghighatnia et al. also found that higher reinforcement loadings significantly increased the surface hardness of natural-reinforced thermoplastic polyurethane systems [63].

The graphs further show that the Shore D hardness values obtained from the three replicate specimens at each reinforcement level are highly consistent. This indicates good repeatability of the hardness measurements and suggests that GHP exhibits an acceptable macroscopic dispersion within the matrix. In addition, the review study by Donato and Mija noted that keratin-based reinforcements can establish sufficient physical interactions with polymer matrices under appropriate processing conditions, leading to consistent improvements in surface-related properties such as hardness [64].

### 3.2. SEM and EDS Analysis of GHP Reinforced Polyurethane Composites

At this stage, high-resolution SEM images were obtained from specimens in six different compositions five different weight ratios and neat horn powder and the reinforcement distributions in the internal structure were examined.

**Figure 11:**
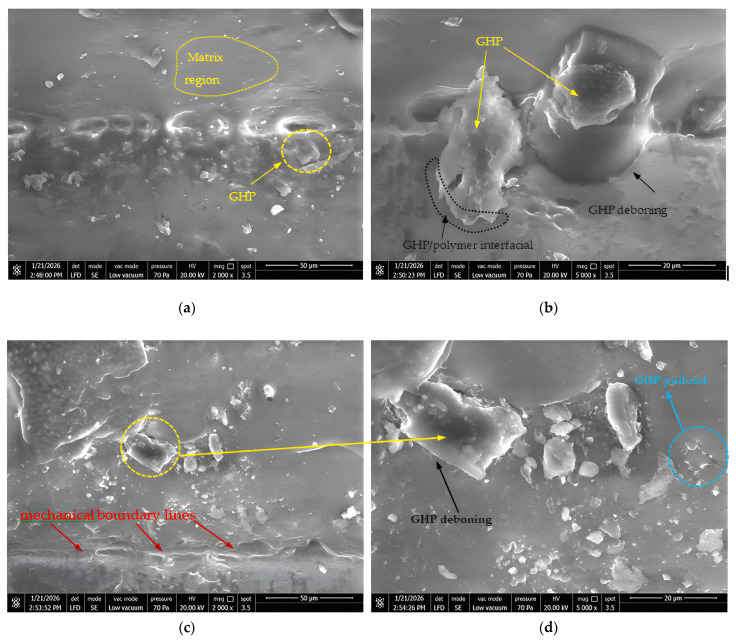

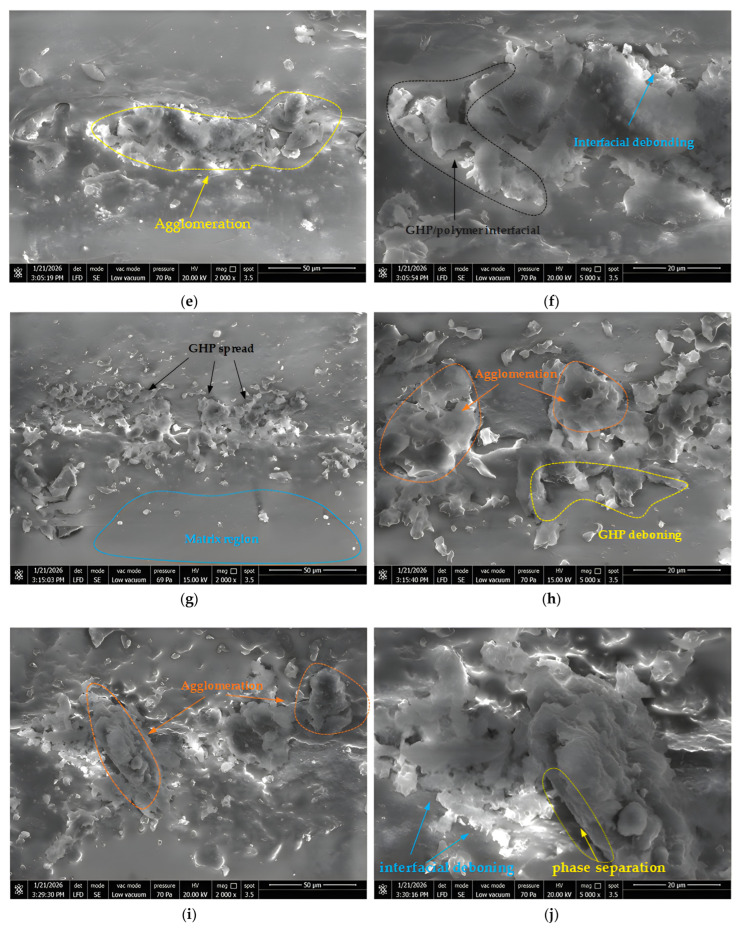

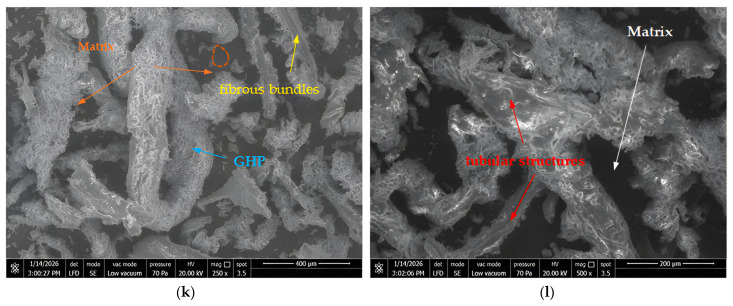
SEM images of the composite materials and goat horn powder: (**a**,**b**) 5 wt.% GHP, (**c**,**d**) 10 wt.% GHP, (**e**,**f**) 15 wt.% GHP, (**g**,**h**) 20 wt.% GHP, (**i**,**j**) 25 wt.% GHP, (**k**,**l**) GHP.

In this part of the study, high-resolution SEM images were obtained from specimens in six different compositions five different weight percentages and neat horn powder and the reinforcement distributions in the internal structure were examined. Figure 11 presents SEM images of the composite materials obtained by combining GHP powders at different weight percentages synthesized with wood-like polyurethane. For each concentration, magnifications of 2000× and 5000× are presented together, side by side.

When the SEM images in Figure 11a,b are examined, they reveal the microstructural characterization of the polymer composite reinforced with 5 wt.% goat horn powder (GHP) in detail. In the image at 5000× magnification (Figure 11b), the characteristic tubular and porous structure of keratin the main component of goat horn as an animal-based biofiller is clearly observed on the particle surface. The irregular and angular geometry of the particles indicates that the keratin fibers fractured in different directions during the grinding process, while this rough structure provides potential for mechanical interlocking with the polymer matrix. However, the thin gaps and distinct boundary lines observed between the keratin particle and the polymer matrix indicate weak interfacial compatibility between the hydrophilic nature of the biofiller and the hydrophobic structure of the matrix. This approach is consistent with the literature compiling interfacial mechanisms reported by Lee et al. [51]. In line with the literature, although low filler contents are often more advantageous, it has been stated by Wrześniewska-Tosik et al. [65] that increasing filler content can increase aggregation/void formation and limit performance. At 2000× magnification (Figure 11a), it is noticeable that at a low filler ratio such as 5%, the particles are generally distributed within the matrix but locally exhibit a tendency toward micro-agglomeration; this demonstrates that higher-energy methods may be needed to ensure homogeneous dispersion of the powder during the mixing process. In parallel with studies by researchers such as Das Lala et al. [66] it is considered that such keratin-based animal reinforcements can increase the hardness and tensile modulus of the composite, but when they cannot form complete chemical bonding with the matrix, they may trigger brittle fracture behavior through the pull-out effect. As a result, these images morphologically confirm that goat horn powder provides a structural reinforcement to the composite structure, but that the interfacial bond must be strengthened to maximize mechanical performance [67].

The SEM micrograph in Figure 11c,d shows mechanical boundary lines on the fracture surface, indicating localized and heterogeneous deformation. The higher-magnification view reveals clear particle–matrix interfacial debonding around GHP particles and pull-out features in some regions. These observations suggest limited interfacial adhesion and indicate that damage initiates preferentially at the interface under load. Consequently, earlier crack initiation and reduced energy absorption can explain the deterioration in mechanical performance.

When Figure 11e,f are examined in detail, the SEM images indicate that, at high GHP contents, the particles are not homogeneously distributed and pronounced agglomeration occurs. These clusters lead to stress concentrations within the matrix, facilitating crack initiation. In addition, the voids and interfacial debonding observed at the GHP–PU interface reveal weak load transfer and show that damage is initiated earlier at the interface. These microstructural features directly explain the deterioration in mechanical properties at filler loadings of ≥15 wt.% GHP.

In the characterization of pure goat horn powder, the observed fibrous bundles and layered keratinous scales, together with the rough surface texture a typical feature of animal horn tissue provide a favorable structure for physical locking (mechanical interlocking) with the polymer matrix. In composites with a 10 wt.% content, it is observed that the particles are homogeneously distributed within the matrix, that the polymer phase fully wets the reinforcement elements and maintains matrix continuity, and that stress transfer is optimized [68].

However, when the reinforcement content increases to 15%, interfacial debonding and distinct boundary lines (phase separation) begin to form due to the incompatibility between the hydrophilic nature of keratin and the hydrophobic structure of the matrix. At 20%, along with the tendency of particles toward agglomeration, stress concentration regions form within the matrix, and deep voids appear in the specimen as a result of particle pull-out [69]. At the highest reinforcement content of 25%, matrix saturation occurs because the binding capacity of the polymer matrix for the particles is exceeded; this leads to extensive void formation and causes the material to gain a brittle fracture surface character and lose its structural integrity [70]. Overall, it is concluded that goat horn powder provides successful reinforcement up to the 10–15% threshold, whereas loadings above this ratio adversely affect mechanical performance due to agglomeration and interfacial weaknesses.

This microstructural evidence (i.e., the onset of interfacial discontinuities and particle clustering from ≥15 wt.% and the more pronounced agglomeration/void formation at 20–25 wt.%) directly supports the mechanical trends reported in Section 3.1. Specifically, the agglomerate-rich regions and interfacial defects act as stress concentrators and reduce effective load transfer, which is consistent with the observed decreases in tensile/flexural performance and toughness at ≥15 wt.% GHP.

The SEM images show in Figure 11g,h that GHP is distributed in the matrix, but the dispersion becomes locally non-uniform, with particle-rich zones and matrix-dominant regions. At higher magnification, pronounced agglomerates are evident, indicating particle clustering rather than a fully homogeneous distribution. Interfacial debonding features around GHP particles suggest limited particle–matrix adhesion and inefficient load transfer. These microstructural defects can act as stress concentrators, promoting early crack initiation and contributing to the reduction in mechanical performance.

The presented SEM images illustrate in Figure 11i,j; the fracture surface morphology of the investigated polymer composite material. When examining the micrograph on the left, it is observed that the filler particles are not homogeneously distributed within the matrix, forming distinct agglomerations. In the higher magnification image on the right, interfacial debonding and phase separations, which are indicative of a weak bond between the matrix and the filler material, are clearly detected. These structural defects indicate poor interfacial interaction between the matrix and the filler, carrying the potential to negatively affect the overall mechanical performance of the material.

The morphological examination of pure goat horn powder (Pure Goat Horn Powder GHP) in Figure 11k,l clearly reveals the complex and hierarchical organization of the animal keratin structure. The fibrous bundles and layered keratinous scales observed in the SEM images represent the dense stacking of protein fibers formed during the natural growth process of the material. The tubular structures and micro-porosity observed on particle surfaces and cross-sections confirm the characteristic “voided” architecture of keratin tissue, and this porous structure provides a large surface area that enables physical locking (mechanical interlocking) with the polymer matrix. The rough surface morphology and lamellar structure of the particles provide a texture that may allow effective stress transfer between the matrix and the reinforcement under mechanical loading. As emphasized by Kumar and Boopathy [15], this natural microstructure of goat horn has the potential to increase the stiffness and impact resistance of biocomposites as a sustainable and high-performance alternative to synthetic fibers.

## 4. Conclusions

In this study, goat horn powder (GHP)–reinforced wood-like polyurethane (PU) composites were investigated in a multidimensional manner by jointly evaluating microstructure (SEM/EDS) and mechanical tests (tensile, three-point bending, Charpy impact, Shore D). A prominent original aspect of this study is the SEM/EDS investigation, which establishes a direct relationship between mechanical behavior and reinforcement distribution, interfacial adhesion, agglomeration, and crack-initiating defects.

In natural particle-reinforced polymer composites, performance largely depends on the reinforcement–matrix interface and dispersion homogeneity. As emphasized by Fu et al. [52], particle size, loading ratio, and interfacial adhesion are decisive in stress concentration, early damage initiation, and embrittlement. Consistent with this, our results indicate an “effective reinforcement” threshold at low GHP contents (5–10 wt.%), where particles partially hinder crack propagation and contribute to load bearing. However, at higher contents (≥15 wt.%), mechanical performance in several metrics decreases. This common “defect-like” behavior in particle-reinforced systems is due to the disruption of matrix continuity, increased interfacial voids, and agglomeration acting as crack initiators, leading to more brittle fracture mechanics [52].

Tensile and flexural results demonstrated this transition clearly. The addition of 5 wt.% GHP increased the average maximum tensile stress by 12.89%. Conversely, flexural stress experienced a reduction compared to the neat polymer (26.59 MPa), dropping to 21.08 MPa at 5 wt.% and decreasing by 66.23% at 25 wt.%. In both tests, increasing GHP content markedly reduced overall ductility evident from the significant drops in elongation at break, toughness (stress–strain area), and maximum deflection.

Impact and hardness properties, however, exhibited different trends. Energy dissipation improved dramatically at lower reinforcement levels, likely due to mechanisms such as crack deflection, microcrack formation, and energy absorption via interfacial friction. Similar to findings by El-Shekeil et al. [60] in TPU-based systems, impact performance eventually decreased at higher contents. Meanwhile, Shore D hardness increased systematically across all reinforcement ratios. Starting from 52–54 Shore D for neat PU, the highest values were obtained at the 20–25 wt.% levels. This localized stiffening is consistent with the hierarchical, lamellar structure of α-keratin in goat horns, affirming its potential as an “engineering material” when paired with polymers, as detailed by Tombolato et al. [10].

Consequently, regarding the effect of animal-waste-derived GHP on PU-matrix composites, the core findings are summarized as follows:

- Functional Reinforcement & Breakthrough Impact Performance: GHP acts as a functional reinforcing element, particularly for impact resistance. A 140% increase in impact strength (7.38 kJ/m^2^) was achieved with 10 wt.% GHP compared to the neat PU (3.05 kJ/m^2^). This demonstrates significant potential for engineering applications exposed to sudden loading (e.g., protective panels or automotive interior components).
- Critical Threshold Values: The optimal reinforcement threshold is 5 wt.% for tensile strength and 10 wt.% for impact strength. Loadings above these ratios induce microstructural agglomerations that negatively affect these specific mechanical metrics.
- Hardness and Stiffness Characteristics: While GHP addition reduces overall ductility, it systematically improves Shore D hardness and stiffness up to the maximum tested reinforcement ratio of 25 wt.%.
- Contribution to Sustainability: Converting waste goat horn into a value-added engineering material both supports environmental waste management and introduces a novel natural reinforcement source to the biocomposite literature.

For future studies, the following points are recommended:

- Interfacial Improvement: To prevent the mechanical decline observed at reinforcement ratios of 15 wt.% and above, chemical surface treatments such as silanization can be applied to GHP particles to enhance matrix–reinforcement adhesion.
- Size Analysis: The effects of sub-100 µm (nano-sized) GHP particles on dispersion homogeneity and mechanical properties should be investigated.
- Environmental Resistance: The material’s moisture absorption capacity and aging performance at different temperatures should be examined to determine its durability in outdoor applications.

## Figures and Tables

**Table 1:** Percentage (%) elemental contents of goat horn.

Main Elements	C	O	N	S	Si
Trace Elements	K	Na	Cl	P	Al

**Table 2:** Mechanical properties of goat horn and wood-like polyurethane materials.

Materials	Density ρ (g/cm^3^)	Young’s Modulus E	UTS MPa	Hardness Shore D	References
Polyurethane	1081	4.66 GPa	8.31	53	[34,35]
Goat horn powder	0.9–1.2	22.1 MPa	95	77	[6,16,36,37,38]

**Table 3:** Composition Mixtures.

S/N	Goat Horn Powder (wt.%)	Polyurethane (wt.%)	Sample Label
1	0	100	%0 GHP
2	5	95	%5 GHP
3	10	90	%10 GHP
4	15	85	%15 GHP
5	20	80	%20 GHP
6	25	75	%25 GHP

## Data Availability

The raw data supporting the conclusions of this article will be made available by the authors on request.
